# Inhibitory Effects of Baicalin on the Expression and Activity of CYP3A Induce the Pharmacokinetic Changes of Midazolam in Rats

**DOI:** 10.1155/2013/179643

**Published:** 2013-04-24

**Authors:** Xin Tian, Zhen-Yu Cheng, Han Jin, Jie Gao, Hai-Ling Qiao

**Affiliations:** Department of Clinical Pharmacology, School of Medicine, Zhengzhou University, Daxue Road 40, Zhengzhou, Henan 450052, China

## Abstract

Baicalin, a flavonoid compound isolated from *Scutellaria baicalensis*, has been shown to possess antiinflammatory, antiviral, antitumour, and immune regulatory properties. The present study evaluated the potential herb-drug interaction between baicalin and midazolam in rats. Coadministration of a single dose of baicalin (0.225, 0.45, and 0.90 g/kg, i.v.) with midazolam (10 mg/kg, i.v.) in rats resulted in a dose-dependent decrease in clearance (CL) from 25%  (*P* < 0.05) to 34%  (*P* < 0.001) with an increase in AUC_0−*∞*_ from 47%  (*P* < 0.05) to 53%  (*P* < 0.01). Pretreatment of baicalin (0.90 g/kg, i.v., once daily for 7 days) also reduced midazolam CL by 43%  (*P* < 0.001), with an increase in AUC_0−*∞*_ by 87%  (*P* < 0.01). Multiple doses of baicalin decreased the expression of hepatic CYP3A2 by approximately 58%  (*P* < 0.01) and reduced midazolam 1′-hydroxylation by 23%  (*P* < 0.001) and 4′-hydroxylation by 21%  (*P* < 0.01) in the liver. In addition, baicalin competitively inhibited midazolam metabolism in rat liver microsomes in a concentration-dependent manner. Our data demonstrated that baicalin induced changes in the pharmacokinetics of midazolam in rats, which might be due to its inhibition of the hydroxylation activity and expression of CYP3A in the liver.

## 1. Introduction

**Figure 1:**
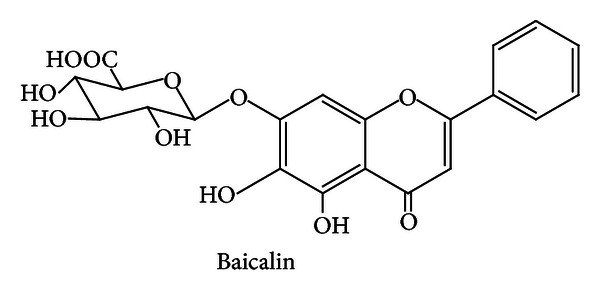
Chemical structure of baicalin.

Baicalin (5, 6-Dihydroxy-flavone-7-O-glucuronide, (Figure 1)) is the major bioactive constituent of *Radix scutellariae *(Huang-Qin in Chinese), which is widely used in eastern and western medicine [1, 2]. As a monomer, baicalin is also commonly used to treat hepatitis patients in China in combination with other drugs. Baicalin has been reported to possess a multitude of pharmacological activities, including antioxidant [3], antiproliferative [4], antiviral [5], antiinflammatory [6], and liver protective [7, 8] properties. Baicalin is the main bioactive constituent of approximately 100 types of Chinese medicine and is used as phytochemical marker for their quality control in Chinese pharmacopoeia [2, 9]. 

Drug-drug interactions (DDIs) are thought to be an important factor in severe adverse drug reactions. The inhibition of the drug-metabolising enzyme cytochrome P450 (CYP) is known to participate in this type of interaction. The CYP3A subfamily is by far the most abundant of all the human CYP isoforms [10] and catalyses the metabolism of nearly 60% of clinical drugs [11]. Therefore, DDIs involving the inhibition of CYP3A are generally considered to be undesirable as they may manifest as unwanted side effects for drugs with a narrow therapeutic window [12, 13]. Furthermore, recent studies [14, 15] revealed that not only chemical drugs but also natural products such as herbs may inhibit CYP3A activity. The widespread use of baicalin as alternative or complementary medicine has led to increasing concerns with respect to potential herb-drug interactions through its effects on enzymatic pathways.

Recent studies showed that baicalin [16] and other main bioactive constituents of *Radix scutellariae* [15] such as baicalein [17, 18] and wogonin [17] have significant inhibitory effects on the metabolism of clinical drugs. Lai et al. [16] reported that oral administration of baicalin significantly increased area under the plasma concentration-time curve (AUC) of cyclosporine in rats. Baicalein significantly enhanced the oral bioavailability of nimodipine [18]. Treatment with baicalein and wogonin resulted in the decreases in the activity and expression of CYP3A in mice [17]. Although reports have demonstrated that baicalin could inhibit the activity of CYP3A, the mechanisms underlying these interactions between CYP3A and baicalin are not well characterised. 

Midazolam (MDZ) is a short-acting benzodiazepine used clinically for conscious sedation. MDZ is metabolised to 1′-hydroxymidazolam and 4-hydroxymidazolam by CYP3A4 and CYP3A5 in humans [19–21] and by CYP3A1 and CYP3A2 in rats [22, 23]. Importantly, CYP3A2 plays the primary role in MDZ metabolism in rats [22, 24]. MDZ is recommended by the FDA as a probe used to determine CYP3A4/5 activity *in vitro* and *in vivo* studies, and it is the most commonly used probe of CYP3A activity in rats and humans [25–28]. In this study, the effects of baicalin on the pharmacokinetics of MDZ were evaluated in rats treated with single and multiple doses of baicalin. To expand on the *in vivo* results, the effects of baicalin on the activity and expression of CYP3A in the rat liver were examined *in vitro* and *ex vivo*. 

## 2. Materials and Methods

### 2.1. Drugs and Materials

MDZ injection was purchased from Nhwa Pharma. Corporation (Jiangsu, China). 1′-Hydroxymidazolam was obtained from Cerilliant Co. (Austin, TX, USA). 4-Hydroxymidazolam was obtained from Sigma Chemical Co. (St. Louis, MO, USA). NADPH was purchased from Roche Co. Ltd (Switzerland). Diazepam injection was obtained from Tianjin Jin Yao Amino Acid Co., Ltd. (China). Baicalin (purity ≥ 98.5%) was the kind gift of the Henan Provincial Institute of Food and Drug Control. All other reagents were high-performance liquid chromatography (HPLC) grade and commercially available.

### 2.2. Animals

Male Sprague-Dawley rats (250–300 g) were obtained from the Laboratory Animal Centre of Henan Province (Henan, China) and maintained in a temperature-controlled environment with a 12 h light-dark cycle. Animals had free access to standard laboratory food and tap water. All experiments were performed after approval of the Zhengzhou University Ethics Committee for Animal Care and Use.

### 2.3. Effects of Baicalin on the Pharmacokinetics of MDZ in Rats

The effects of baicalin on the pharmacokinetics of MDZ (10 mg/kg, i.v.) were studied at three different doses (0.225, 0.45, and 0.90 g/kg, i.v.). The solution for injection was prepared by dissolving 250 mg BG in 50 mL of Na_2_HPO_4_ (0.2 M) and adjusting to pH7.4 with citric acid (0.1 M).

In the first study, 9 rats were randomly divided into 3 groups (*n* = 3, each), and the order of the baicalin doses followed a Latin-Square design (saline, 0.225 g/kg, 0.45 g/kg) with a 3 day wash-out period between treatments. MDZ was administered immediately following the injection of baicalin or saline via tail vein. On each of the three occasions, blood samples (500 **μ**L) were collected before baicalin administration and at 0, 0.083, 0.17, 0.33, 0.67, 1, 1.5, and 2 h after MDZ administration by orbital bleeding via heparinised capillary tubes. The samples were centrifuged at 4,500 ×g for 10 min at 4°C, and separated plasma was frozen at −80°C prior to analysis. 

In another experiment, 16 rats were randomly divided into saline group and baicalin group (*n* = 8, each). First, the inhibition of a single dose (0.90 g/kg, i.v.) of baicalin on MDZ pharmacokinetics was studied in a randomised crossover study in the baicalin group. After the single-dose study, the same rats were included in a multiple doses of investigation and were treated with baicalin (0.90 g/kg, i.v.) once daily for 7 days. On day 8, MDZ was given to the rats after the last dose of baicalin. The procedures for MDZ administration and sampling were consistent with those described previously. All 16 rats from both groups were sacrificed by cervical dislocation 24 h after the last dose of baicalin, and the liver was excised. Liver microsomes were prepared by calcium aggregation as described previously [29]. 

Briefly, to obtain the postmitochondrial supernatant, liver tissue homogenate was centrifuged at 4°C (12,500 g, 20 min). The addition of CaCl_2_ (8.8 mM final concentration) to the supernatant allowed the complete sedimentation of the microsomes at 4°C (25,000 g, 20 min). The pellet was washed by resuspending in an excess volume of homogenisation solution and then was resedimented at 25,000 g for 20 min. The resultant pinkish opalescent microsomal pellet was suspended in 0.25 M sucrose phosphate buffer and stored at −80°C for subsequent enzyme activity assays.

### 2.4. Determination of Plasma MDZ Concentration

The HPLC method used for the analysis of MDZ was modified from Jurica et al. [30]. In brief, 10 *μ*L of diazepam (0.09 mg/mL) was used as the internal standard, and 90 *μ*L NaOH solution (0.1 M) and 3 mL ether were added to 100 *μ*L of the plasma sample. The mixture was vortexed for 3 min and centrifuged at 3000 rpm for 10 min. Two and a half millilitres of the organic phase was transferred into a clean glass tube and dried under nitrogen at 45°C. The residue was reconstituted with 100 *μ*L of mobile phase, and 40 *μ*L was injected onto the HPLC system (Aligent 1100 Series) with a UV detector set at 220 nm for the analysis. The C18 column (4.6 mm × 250 mm; Dikima Technologies) was set at 25°C. The flow rate was 1.0 mL/min, and the mobile phase consisted of 100 mM ammonium acetate aqueous solution (pH 4.0), acetonitrile, and methanol in a ratio of 34 : 13 : 53 (v/v/v). The method was linear over 0.25–8.14 mg/L.

### 2.5. Determination of Plasma Baicalin Concentration

The plasma concentration of baicalin was measured by HPLC with UV detection at 278 nm, as described previously [31] with minor modifications. Each plasma sample (25 *μ*L) was added to 100 *μ*L of methanol. After vortexing for 60 s and centrifugation for 10 min at 4°C (15,000 g), the supernatant (5 *μ*L) was injected into a Diamonsil C18 column (4.6 mm × 200 mm; Dikma Technologies) for analysis. The mobile phase consisted of 10 mM potassium phosphate buffer (pH 5.0): methanol (47 : 53), and the flow rate was 1.0 mL/min. The method was linear over 5.86–3000 mg/L. 

### 2.6. Effects of Multiple Doses of Baicalin on the Activity and Expression of CYP3A in the Liver

Rat liver microsomes (RLMs) prepared 24 h after the last dose of one week of baicalin or saline treatments were used in the *ex vivo* study to determine MDZ hydroxylation activity and CYP3A expression. 

#### 2.6.1. MDZ Hydroxylation Activity Assay

The incubation mixtures (total volume 0.2 mL) contained MDZ (3.125–200 **μ**M), microsomal protein (0.25 mg/mL), phosphate buffer (potassium dihydrogen phosphate, 100 mM, pH 7.4), MgCl_2_ (3 mM), and EDTA (0.1 mM). The reaction was initiated by the addition of *β*-nicotinamide adenine dinucleotide phosphate (NADPH, 1 mM) and terminated by adding ice-cold acetonitrile (20 *μ*L). The mixtures were vortexed and centrifuged (15,000 g, 10 min). The supernatant (20 *μ*L) was injected into the HPLC system. Calibration was performed in the range from 0.45 to 28.82 *μ*M for 1′-hydroxymidazolam and 4-hydroxymidazolam. 

#### 2.6.2. Measurement of CYP3A2 Expression

CYP3A2 plays a key role in the appearance of 1′-hydroxymidazolam and 4-hydroxymidazolam [22, 24], and western blotting analysis was carried out to investigate the effect of multiple doses of baicalin on its expression. Rat hepatic microsome proteins (12 *μ*g) were resolved on an 8% or 12% SDS-polyacrylamide gel and transferred to a polyvinylidene difluoride membrane (Millipore, USA). Anti-CYP3A2 antibody (1 : 10000, Abcam Ltd., Hong Kong) and anti-GAPDH antibody (1 : 500, CWBIO, Beijing, China) were incubated overnight at 4°C, and then horseradish peroxidase-conjugated secondary antibody (1 : 5000, Beijing Biosynthesis Biotechnology Co., Ltd., China) was incubated for 1 h at room temperature. Chemiluminescence was detected with enhanced chemiluminescence substrate (Beyotime Institute of Biotechnology, China) on X-ray films. The band was then scanned, and the intensity was quantified using the Image J software (NIH). 

### 2.7. Determination of *K*_*i*_ of Baicalin against MDZ Metabolism in RLMs

RLMs were prepared from 10 untreated male Sprague-Dawley rats. MDZ was incubated at concentrations equivalent to 0.5 × *K*_*m*_,  *K*_*m*_,   and  2 × *K*_*m*_, with a range of baicalin concentrations (0, 20, 40, 80, 160, and 320 *μ*M). Incubations were performed as described for the MDZ hydroxylation activity study. 

### 2.8. Data Analysis

Michaelis-Menten enzyme kinetics data were fit by nonlinear regression analysis using GraphPad Prism 5 (GraphPad Software Inc., CA, USA). The mechanism of inhibition was determined by visual inspection of the data using Dixon (1/v versus [I]) and Lineweaver-Burke (1/v versus 1/[S]) plots. The *K*_*i*_ was obtained using the secondary Lineweaver-Burk plot. 

The plasma concentration versus time data were assessed via noncompartmental analysis using the DAS 2.0 package (version 2.0 pharmacokinetic software; Chinese Pharmacological Assn., Beijing, China). The area under the plasma concentration-time curve (AUC) was calculated according to the trapezoidal rule. The peak plasma concentrations (*C*_max⁡_) were obtained from the actual data. Clearance (CL) is defined as the total clearance calculated by CL = *D*/AUC as the volume of plasma from which the drug is totally removed in unit time by all elimination processes in the body. The *C*_max⁡_, AUC, and CL data were analysed by the paired *t*-test. The results are expressed as the mean ± SD. A value of *P* < 0.05 was considered to be statistically significant. All statistical analyses were performed with SPSS 17.0 for Windows.

## 3. Results

### 3.1. The Pharmacokinetic Changes of MDZ Induced by Baicalin in Rats

#### 3.1.1. The Pharmacokinetics of Baicalin

**Figure 2:**
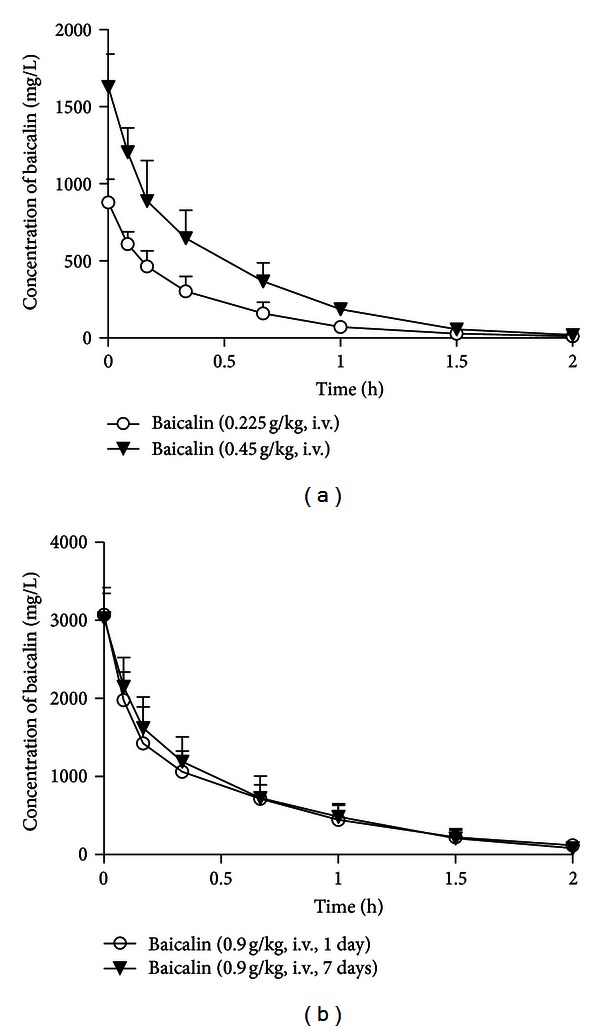
The plasma concentration-time profiles of baicalin. Rats received intravenous administration of baicalin (a) at a single dose of 0.225 or 0.45 g/kg (*n* = 9); (b) at a dose of 0.90 g/kg once for 1 day and the same dose for 7 days (*n* = 8). The results are the mean ± SD of the indicated number of rats.

The mean plasma concentration-time profiles of baicalin after its intravenous administration (0.225, 0.45, and 0.90 g/kg) are illustrated in Figure 2. The key pharmacokinetic parameters of baicalin are summarised in Table 1. The plasma concentrations of baicalin declined rapidly, with a mean *t*_1/2_ approximately 0.4 h (0.32 h to 0.49 h). The observed approximately linear increases in systemic exposure indicated that the elimination of baicalin was characterised by linear pharmacokinetics (*r* = 0.9389). The pharmacokinetic parameters of a single dose of baicalin (0.90 g/kg) were similar to those of once dose daily administration for 7 days (Table 1).

#### 3.1.2. Single Dose of Baicalin

**Figure 3:**
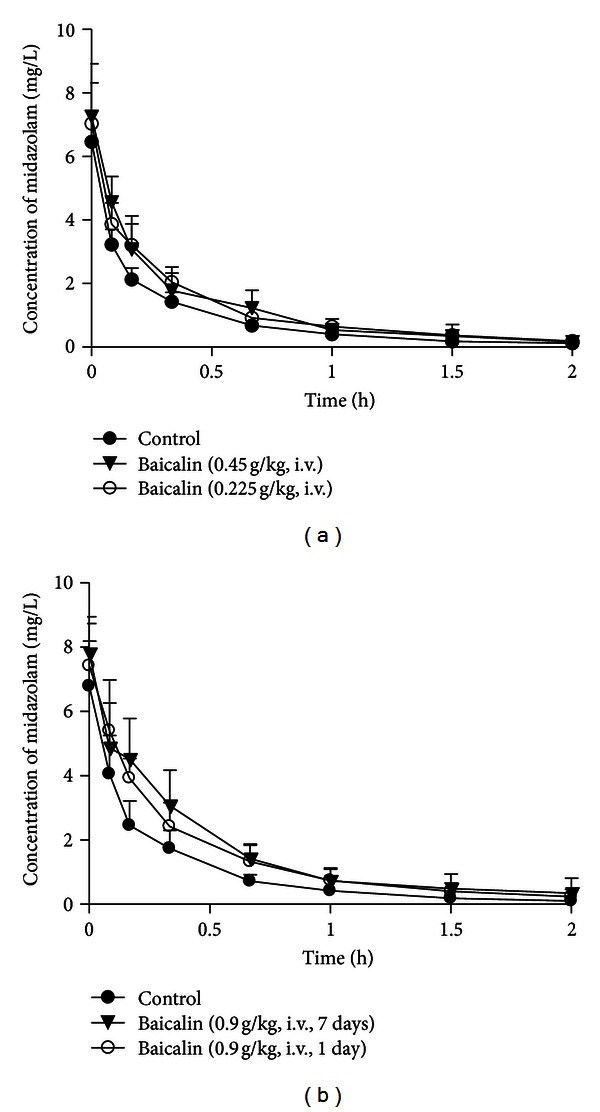
The plasma concentration-time profiles of MDZ (10 mg/kg, i.v.) after treatment with baicalin in rats. Animals received MDZ together with (a) baicalin at a single dose of 0.225 g/kg (*n* = 8) or 0.45 g/kg (*n* = 9); (b) baicalin (0.90 g/kg, i.v.) once for 1 day and the same dose for 7 days (*n* = 8). The results are the mean ± SD of the indicated number of rats.

**Figure 4:**
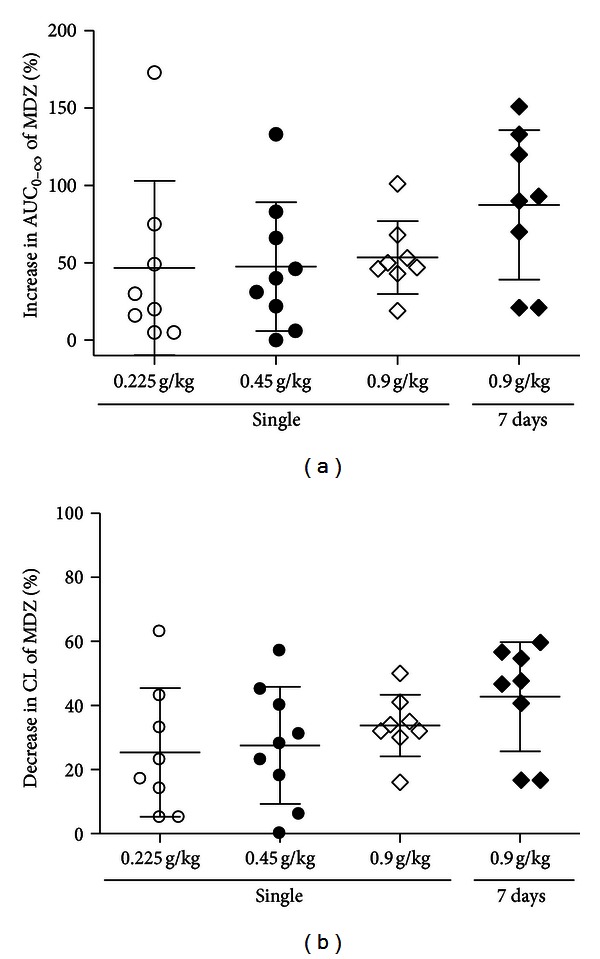
The increase in AUC_0–*∞*_ (a) and the decrease in CL (b) of MDZ (10 mg/kg, i.v.) after treatment with baicalin at a single dose of 0.225 g/kg (*n* = 8) or 0.45 g/kg (*n* = 9) or at a dose of 0.90 g/kg once for 1 day and the same dose for 7 days (*n* = 8).

The mean MDZ plasma concentration-time profiles after administration of MDZ (10 mg/kg, i.v.) with baicalin (0.225, 0.45, and 0.90 g/kg, i.v.) are illustrated in Figure 3. The pharmacokinetic parameters of MDZ in the baicalin-treated groups are shown in Tables 2 and 3. The results indicated that baicalin treatment increased the AUC_0–*∞*_ in a dose-dependent manner, by 47% (*P* < 0.05), 47% (*P* < 0.01), and 53% (*P* < 0.01), in the low-, median-, and high-dose groups, respectively, with corresponding decreases in CL by 25% (*P* < 0.05), 28% (*P* < 0.01), and 34% (*P* < 0.001), respectively. In contrast, the values of other parameters such as the volume of distribution (*V*) were not significantly altered after baicalin treatment. A dose-effect relationship did not exist (Figure 4).

#### 3.1.3. Pretreatment with Multiple Doses of Baicalin

The plasma concentration-time profiles of MDZ after baicalin treatment (0.90 g/kg, once daily for 7 days, i.v.) are shown in Figure 3(b). The plasma MDZ concentrations increased when it was coadministered with baicalin. The *C*_max⁡_, AUC_0–*t*_, and AUC_0–*∞*_ values in rats those received multiple doses of baicalin increased by 27% (*P* < 0.05), 86% (*P* < 0.01), and 87% (*P* < 0.01), respectively (Table 2). Meanwhile, multiple doses of baicalin also decreased the CL and V values by 43% (*P* < 0.001) and 40% (*P* < 0.01), respectively. 

Compared with a single dose of baicalin (0.90 g/kg, i.v.), the *C*_max⁡_,  AUC_0–*t*_  AUC_0–*∞*_, and CL values did not significantly change in the group that received multiple doses of baicalin (Table 2, Figure 4). 

#### 3.1.4. Individual Variability in MDZ Pharmacokinetic Changes

**Figure 5:**
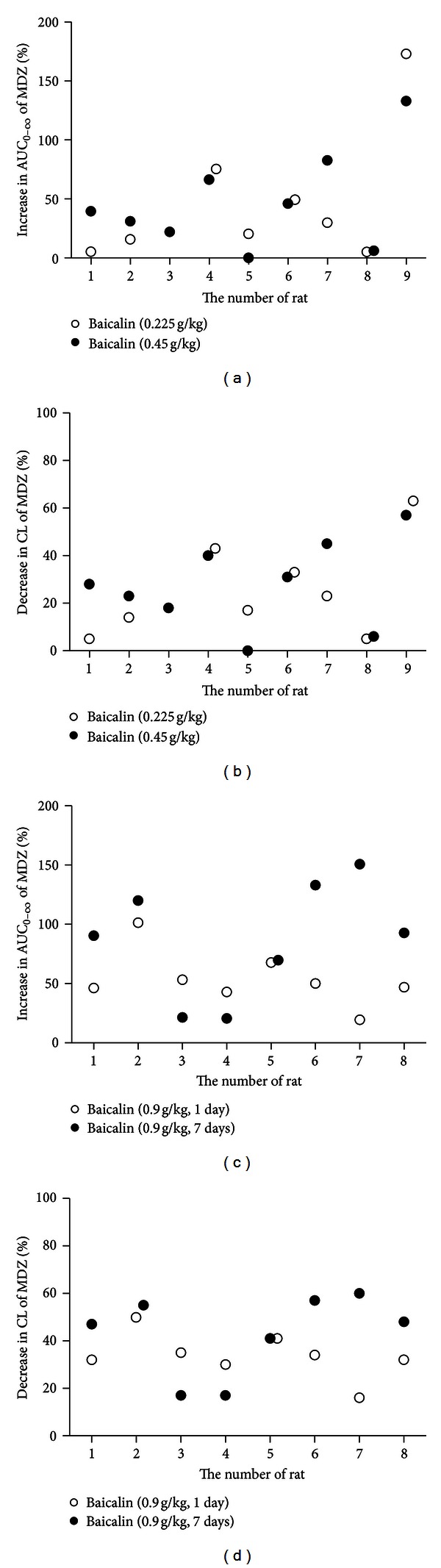
The interindividual differences in the extent of changes in the AUC and CL of MDZ by baicalin. The changes in the AUC_0–*∞*_ ((a), (c)) and CL ((b), (d)) of MDZ (10 mg/kg, i.v.) in rats after treatment with baicalin at a single dose of 0.225 g/kg (*n* = 8) or 0.45 g/kg (*n* = 9) or at a dose of 0.90 g/kg once for 1 day and the same dose for 7 days (*n* = 8).

The variation of the baicalin inhibition between individuals is shown in Figure 5. The increase in the AUC_0–*∞*_ of MDZ by low-, median-, and high doses of baicalin ranged from 5% to 173%, 0 to 133%, and 19% to 101%, respectively (Figures 5(a) and 5(c)). The increase in the AUC_0–*∞*_ of the group that received multiple doses of baicalin ranged from 21% to 151% for MDZ (Figure 5(c)). Corresponding CL changes were also observed (Figures 5(b) and 5(d)). The data showed that the differences in the extent of the increase in the AUC_0–*∞*_ of MDZ were over 173-fold in rats and indicated large interindividual variability in MDZ-baicalin interactions.

#### 3.1.5. Relationship between Pharmacokinetics of Baicalin and MDZ

There were no significant concentration-effect relationships between changes in the MDZ pharmacokinetic parameters and the pharmacokinetics of baicalin (data not shown).

### 3.2. Inhibitory Effects of Baicalin on the Activity and Expression of CYP3A in the Liver

MDZ hydroxylation activities mediated by CYP3A in baicalin-treated (0.90 g/kg, once daily for 7 days) RLMs are shown in Table 4. Multiple doses of baicalin resulted in a decrease in CL_int⁡_ by 23% (*P* < 0.001) and 21% (*P* < 0.01) for 1′-hydroxymidazolam and 4-hydroxymidazolam, respectively, with a corresponding decrease in *V*_max⁡_ by 26% (*P* < 0.01) and 28% (*P* < 0.01), respectively. The data suggested that multiple doses of baicalin inhibited the activity of CYP3A in RLMs.

**Figure 6:**
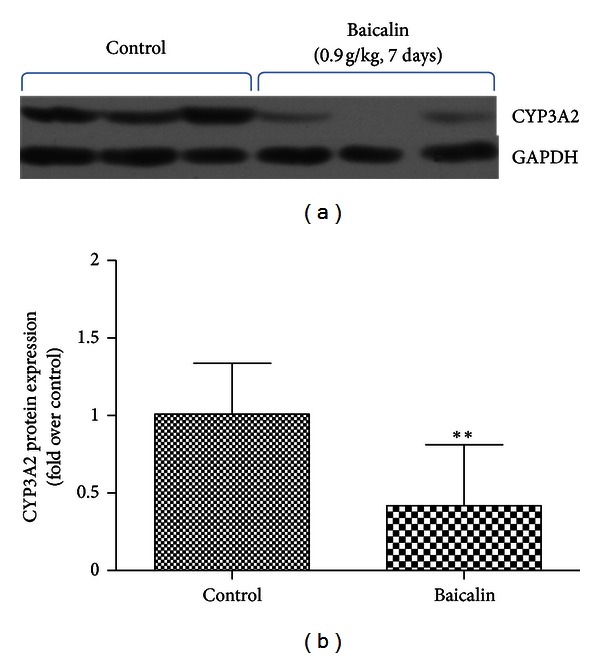
The effects of baicalin (0.90 g/kg, once daily for 7 days) on the expression of CYP3A2 in the rat liver. (a) Western blotting results. (b) Statistical analysis of western blotting results. CYP3A2 expression was measured using specific anti-rat CYP3A2 antibodies. GAPDH was used as the control for the normalisation of CYP3A2 density. Bars represent the mean ± SD of the fold change relative to the values in the control group (*n* = 8). Statistical significance is indicated as **P < 0.01, compared to control.

The effect of multiple doses of baicalin on the expression of CYP3A2 was measured by western blotting. The CYP3A2 antibodies specifically recognised a protein band of 58 kDa.  Figure 6 showed that the intensity of the CYP3A2 expression in baicalin-treated rats was 42% of that observed in control rats (*P* < 0.01). The results indicated that multiple doses of baicalin inhibited the expression of CYP3A2 in rat liver.

### 3.3. Inhibitory Effects of Baicalin on MDZ-Hydroxylation Activity in RLMs

**Figure 7:**
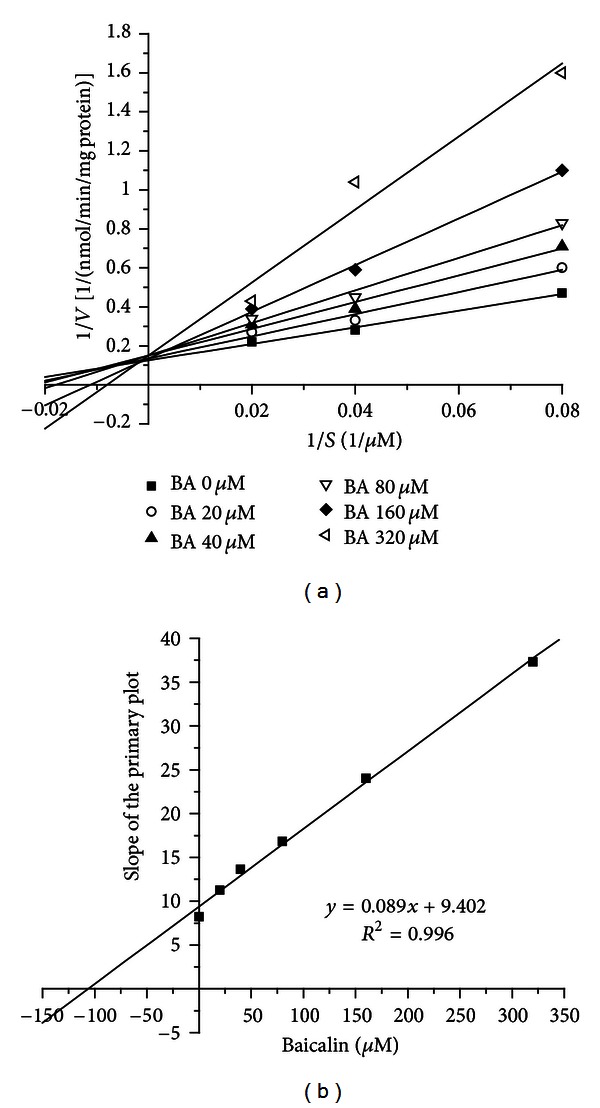
Reversible inhibition of CYP3A by baicalin in rat liver microsomes. (a) Lineweaver-Burk plot of the inhibitory effect of baicalin on CYP3A-mediated MDZ hydroxylation activity. The reciprocal of MDZ disappearance is plotted versus the reciprocal of MDZ concentration in the presence and absence of the inhibitor baicalin (0, 20, 40, 80, 160, and 320 **μ**M). The “*v*” represents the velocity of the disappearance of MDZ. MDZ was used at concentrations of 12.5, 25, and 50 **μ**M. (b) Secondary plot of the slopes from the Lineweaver-Burk plot versus baicalin concentrations. BA: baicalin.

Interestingly, the inhibition of baicalin on the appearance of 1′-hydroxymidazolam in RLMs was minimal, and the *K*_*i*_ could not be calculated based on the results of the inhibitory study. On the other hand, baicalin obviously inhibited the appearance of 4-hydroxymidazolam. Thus, the disappearance of MDZ was used as the index of the inhibitory effect of baicalin on MDZ hydroxylation activity. The Lineweaver-Burk plots for the disappearance of MDZ in RLMs are shown in Figure 7. The Lineweaver-Burk plots (Figure 7(a)) exhibit a series of lines converging on the *y*-axis (the inverse of the substrate (MDZ) concentration), suggesting competitive inhibition of MDZ metabolism by baicalin in RLMs. The *K*_*i*_ value was calculated from the second plot (Figure 7(b)) of the slopes from the Lineweaver-Burk plot versus the concentrations of baicalin and found to be 105.6 *μ*M (47 mg/L).

## 4. Discussion

Clinical and experimental evidence of DDIs has suggested that changes in the pharmacokinetic and pharmacodynamic properties of administered drugs can potentially enhance toxicity and/or attenuate drug efficacy [32]. This is especially important for subjects suffering from chronic infections, such as hepatitis patients, who are dependent on long-term antiretroviral treatment. Baicalin has been used as a phytochemical marker for quality control of *Radix scutellariae* and many other traditional Chinese medicines in Chinese pharmacopoeia [2, 9]. A combined therapy composed of monomeric baicalin and antiretrovirals such as adefovir dipivoxil is also used to treat hepatitis B patients in China. MDZ is used clinically for conscious sedation and commonly employed in preanesthesia. Baicalin is widely included in clinical trials which used to treat many kinds of diseases. Therefore, herb-drug interactions between baicalin and MDZ would appear in therapy. In this study, the interaction between baicalin and MDZ was investigated using a self-control rat model to avoid interindividual variability.

After the intravenous coadministration of MDZ and a single dose of baicalin (0.225, 0.45, and 0.90 g/kg), the AUC of MDZ was greater than that found in rats who did not receive baicalin, possibly due to the slower CL of MDZ (Tables 1 and 2). The slower CL of MDZ in rats was at least partly supported by the slower hepatic CL_int⁡_ for the CYP3A-mediated disappearance of MDZ after treatment with baicalin* in vitro*. After the intravenous administration of both drugs, the *C*_max⁡_ of baicalin in the plasma was 879, 1629, and 3070 mg/L in low-, median-, and high dose baicalin-treated rats, respectively. The *C*_max⁡_ values of baicalin were far higher than the *K*_*i*_ (47 mg/L), so the concentration of baicalin in rats was high enough to inhibit MDZ metabolism. MDZ is metabolised principally by CYP3A4 and CYP3A5 into two primary hydroxylated metabolites (1′-hydroxymidazolam and 4-hydroxymidazolam) [19, 20]. MDZ was also reported to be biotransformed into the same metabolites via CYP3A1 and 3A2 in rats [22, 23]. Therefore, MDZ has been used as an ideal *in vivo* probe to determine CYP3A activity in the rat [25, 33]. The results suggested that the lower CL (greater AUC) of MDZ when the drugs were administered together could be attributable to the inhibition of hepatic metabolism of MDZ by baicalin inhibition of CYP3A1/2. 

Lai et al. [16] found that the oral administration of baicalin significantly increased the absorption of cyclosporine in rats without altering the elimination rate. In our study, baicalin significantly decreased the CL of MDZ in rats when MDZ was administered intravenously immediately after the injection of baicalin. This may be due to the differences in the substrates used in the two studies—MDZ in the present study and cyclosporine in the previous report [16]. Although MDZ and cyclosporine are commonly used as CYP3A probes, Kenworthy et al. [34] indicated that MDZ shows different substrate behaviour compared with cyclosporine and testosterone. 

DDIs are particularly important for patients who are dependent on long-term treatment. Baicalin is commonly used in long-term combined therapy to treat patients with hepatitis in China. To mimic the situation in clinical practice, rats were administered baicalin for seven consecutive days to evaluate the chronic effect of baicalin on the pharmacokinetics of MDZ. The results showed that multiple doses of baicalin significantly decreased the CL (43%) of MDZ with a concomitant increase in the *C*_max⁡_ (27%) and AUC_0–*∞*_ (87%). The reversible inhibition of baicalin has been demonstrated *in vitro*, our data also showed that the slower CL of MDZ could be at least partly supported by the significantly slower CL_int⁡_ of the appearance of 1′-hydroxymidazolam and 4-hydroxymidazolam *ex vivo *(Table 3). The plasma concentrations of baicalin declined rapidly with a mean elimination *t*_1/2_ approximately 0.42 h (Table 1), which is consistent with a recent study [35], so it was clear that baicalin could not be detected in the plasma of rats sacrificed 24 h after the last dose of baicalin (Figure 2(b)). We also demonstrated that baicalin could not be determined in the RLMs of the same rats. Thus, the lower CL_int⁡_ in the *ex vivo* study is attributed to changes in CYP3A activity induced by multiple doses of baicalin. 

Although Li et al. [36] and Hou et al. [37] have reported that baicalin did not affect the expression of CYP3A4 in LS174T cells and CYP3A11 in mice, a previous study [17] showed that the expression level of CYP3A was reduced by baicalein in C57BL/6J mice, with decreases of nifedipine oxidation and erythromycin N-demethylation activities. Baicalin, not baicalein (a glycone of baicalin), has been detected even after oral administration of baicalin in rats [38], so it is speculated that the lower hepatic MDZ hydroxylation activity in our study should be attributed to the lower expression of CYP3A in the rats receiving multiple doses of baicalin. The expression level of CYP3A2 protein was over 128 times higher than that of CYP3A1 in male Sprague-Dawley rats [24], so CYP3A2 may play a decisive role in MDZ hydroxylation activity. Our findings demonstrated that CYP3A2 expression in baicalin-treated rat liver was only 42% of the control (Figure 6). The results indicated that multiple doses of baicalin could inhibit the expression of CYP3A2, and it may be one reason for pharmacokinetic changes in MDZ processing in rats. The results implied that the increase in the systemic exposure of MDZ may lead to an adverse drug reaction in patients receiving long-term treatment of baicalin.

In this study, pharmacokinetic changes were evaluated using an established self-control rat model. We observed that the increase in AUC_0–*∞*_ of MDZ ranged from 0 to 173% after baicalin administration (Figure 5). Large interindividual variations in response to CYP inhibition have also been observed in humans. For example, a 5-fold variation in the extent of increase in the oral AUCs of terfenadine was reported during ketoconazole administration [39]. Significant inter-individual variability was also observed for MDZ grapefruit juice interactions, and the increase in oral AUCs by grapefruit juice inhibition ranged from 26% to 100% for MDZ [40]. Many factors are responsible for the variability in enzyme inhibition, including variability in the inhibitor concentrations and *K*_*i*_ values, susceptibility of drugs to CYP inhibition *in vivo*, genetic variations of the enzyme, and the basal level of enzyme [41, 42]. In future studies, the factors that play key roles in the individual variability of the extent of changes in AUC for MDZ caused by baicalin treatment should be investigated.

## 5. Conclusions 

In conclusion, baicalin significantly enhanced systemic exposure to MDZ (AUC), which might be mainly due to the competitive inhibition of baicalin on CYP3A-mediated MDZ metabolism in the rat liver. Multiple doses of baicalin significantly decreased the activity and expression of CYP3A in the rat liver. The interaction, which was demonstrated *in vivo* and *in vitro* in rats, implies that significant clinical consequences might occur during the concomitant administration of baicalin and MDZ. Thus, patients receiving MDZ should be cautioned against the intake of baicalin/*Radix scutellariae*-derived products to prevent potential adverse drug interactions. 

## Figures and Tables

**Table 1:** Pharmacokinetic parameters of baicalin after intravenous administration in rats.

	Baicalin (0.225 g/kg)	Baicalin (0.45 g/kg)	Baicalin (0.90 g/kg)	
			Single	Multiple^a^
*C*_max⁡_ (mg/L)	879 ± 150	1630 ± 212	3070 ± 350	3030 ± 314
*t*_1/2_ (h)	0.34 ± 0.06	0.32 ± 0.04	0.49 ± 0.13	0.42 ± 0.13
*V* (L/kg)	0.36 ± 0.12	0.16 ± 0.05	0.45 ± 0.10	0.39 ± 0.10
CL (L/h/kg)	0.72 ± 0.15	0.34 ± 0.07	0.65 ± 0.14	0.66 ± 0.16
AUC_0–*t*_ (mg·h/L)	320 ± 66	673 ± 123	1342 ± 230	1374 ± 308
AUC_0–*∞*_ (mg·h/L)	325 ± 67	683 ± 121	1438 ± 286	1441 ± 332

^a^Once daily for 7 days. Rats received intravenous administration of baicalin at a single dose of 0.225 or 0.45 g/kg (*n* = 9) or at a dose of 0.90 g/kg once for 1 day and the same dose for 7 days (*n* = 8). The results were means ± SD of the indicated number of rats.

**Table 2:** Pharmacokinetic parameters of MDZ (10 mg/kg, i.v.) after treatment with a single dose of baicalin (0.225, 0.45 g/kg, i.v.) in rats.

	Control	Baicalin (0.225 g/kg)		Baicalin (0.45 g/kg)	
	Value	Value	Ratio	Value	Ratio
*C*_max⁡_ (mg/L)	6.5 ± 1.0	7.0 ± 1.4	1.10 (0.94 ~ 1.26)	7.3 ± 1.9	1.15 (0.89 ~ 1.41)
*t*_1/2 _ (h)	0.44 ± 0.08	0.51 ± 0.18		0.51 ± 0.16	
*V* (L/kg)	3.6 ± 0.7	3.0 ± 1.0		3.1 ± 1.4	
CL (L/h/kg)	5.9 ± 0.9	4.3 ± 0.9*	0.75 (0.58 ~ 0.92)	4.2 ± 0.8**	0.72 (0.58 ~ 0.87)
AUC_0–*t*_ (mg·h/L)	1.7 ± 0.2	2.3 ± 0.7*	1.42 (1.01 ~ 1.84)	2.4 ± 0.6*	1.44 (1.12 ~ 1.75)
AUC_0–*∞*_ (mg·h/L)	1.7 ± 0.2	2.5 ± 0.8*	1.47 (1.00 ~ 1.94)	2.5 ± 0.6**	1.47 (1.15 ~ 1.79)

The results are means ± SD of 8-9 rats. Ratios are expressed as geometric mean ratio with 90% CI. Significance is indicated as *P < 0.05; **P < 0.01 compared to the control.

**Table 3:** Pharmacokinetic parameters of MDZ (10 mg/kg, i.v.) after treatment with baicalin.

	Control	Baicalin (0.90 g/kg, 1 day)		Baicalin (0.90 g/kg, 7 days)	
	Value	Value	Ratio	Value	Ratio
*C*_max⁡_ (mg/L)	6.3 ± 1.3	7.0 ± 1.2	1.13 (0.92 ~ 1.34)	7.8 ± 0.9*	1.27 (1.03 ~ 1.51)
*t*_1/2_ (h)	0.40 ± 0.10	0.47 ± 0.11		0.44 ± 0.10	
*V* (L/kg)	3.2 ± 0.9	2.5 ± 0.5		1.9 ± 0.4**	
CL (L/h/kg)	5.6 ± 0.7	3.7 ± 0.6***	0.66 (0.58 ~ 0.74)	3.2 ± 0.8***	0.57 (0.43 ~ 0.71)
AUC_0–*t*_ (mg*·*h/L)	1.7 ± 0.2	2.6 ± 0.4**	1.50 (1.34 ~ 1.67)	3.2 ± 0.8**	1.86 (1.48 ~ 2.24)
AUC_0–*∞*_ (mg*·*h/L)	1.8 ± 0.2	2.8 ± 0.5**	1.53 (1.34 ~ 1.73)	3.4 ± 0.9**	1.87 (1.47 ~ 2.28)

Rats received intravenous administration of baicalin at a dose of 0.90 g/kg once for 1 day and the same dose for 7 days. The results are means ± SD of 8 rats. Ratios are expressed as geometric mean ratio with 90% CI. Significance is indicated as *P < 0.05; **P < 0.01; ***P < 0.001 compared to the control.

**Table 4:** Effects of multiple doses of baicalin on MDZ hydroxylation activity in the rat liver.

	Group	*V*_max⁡_ (nmol/min/mgprotein)	*K*_*m*_ (**μ**M)	CL_int⁡_ (*μ*L/min/mg protein)
1′-Hydroxymidazolam	Control	0.528 ± 0.061	18.6 ± 2.4	28.4 ± 1.5
	Baicalin	0.388 ± 0.075**	18.2 ± 5.0	21.8 ± 3.1***
4-Hydroxymidazolam	Control	1.188 ± 0.227	15.4 ± 3.6	77.9 ± 9.3
	Baicalin	0.849 ± 0.143**	14.2 ± 4.0	61.7 ± 11.1**

Rats received intravenous administration of baicalin at a dose of 0.90 g/kg once daily for 7 days. The results are means ± SD of 8 rats. Significance is indicated as **P < 0.01, ***P < 0.001 compared to the control.

## References

[1] Li C, Lin G, Zuo Z (2011). Pharmacological effects and pharmacokinetics properties of Radix Scutellariae and its bioactive flavones. *Biopharmaceutics & Drug Disposition*.

[2] Boyle SP, Doolan PJ, Andrews CE, Reid RG (2011). Evaluation of quality control strategies in *Scutellaria* herbal medicines. *Journal of Pharmaceutical and Biomedical Analysis*.

[3] Gao Z, Huang K, Xu H (2001). Protective effects of flavonoids in the roots of *Scutellaria baicalensis* Georgi against hydrogen peroxide-induced oxidative stress in HS-SY5Y cells. *Pharmacological Research*.

[4] Ikemoto S, Sugimura K, Yoshida N (2000). Antitumor effects of *Scutellariae radix* and its components baicalein, baicalin, and wogonin on bladder cancer cell lines. *Urology*.

[5] Evers DL, Chao CF, Wang X, Zhang Z, Huong SM, Huang ES (2005). Human cytomegalovirus-inhibitory flavonoids: studies on antiviral activity and mechanism of action. *Antiviral Research*.

[6] Lixuan Z, Jingcheng D, Wenqin Y, Jianhua H, Baojun L, Xiaotao F (2010). Baicalin attenuates inflammation by inhibiting NF-*κ*B activation in cigarette smoke induced inflammatory models. *Pulmonary Pharmacology and Therapeutics*.

[7] Xu Y, Feng Y, Li H, Gao Z (2012). Ferric citrate CYP2E1-independently promotes alcohol-induced apoptosis in HepG2 cells via oxidative/nitrative stress which is attenuated by pretreatment with baicalin. *Food and Chemical Toxicology*.

[8] Parajuli P, Joshee N, Rimando AM, Mittal S, Yadav AK (2009). In vitro antitumor mechanisms of various *Scutellaria* extracts and constituent flavonoids. *Planta Medica*.

[9] Wu S, Sun A, Liu R (2005). Separation and purification of baicalin and wogonoside from the Chinese medicinal plant *Scutellaria baicalensis* Georgi by high-speed counter-current chromatography. *Journal of Chromatography A*.

[10] Shimada T, Yamazaki H, Mimura M, Inui Y, Guengerich FP (1994). Interindividual variations in human liver cytochrome P-450 enzymes involved in the oxidation of drugs, carcinogens and toxic chemicals: studies with liver microsomes of 30 Japanese and 30 Caucasians. *Journal of Pharmacology and Experimental Therapeutics*.

[11] Tang W, Stearns RA (2001). Heterotropic cooperativity of cytochrome P450 3A4 and potential drug-drug interactions. *Current Drug Metabolism*.

[12] Vuppugalla R, Zhang Y, Chang S, Rodrigues AD, Marathe PH (2012). Impact of nonlinear midazolam pharmacokinetics on the magnitude of the midazolam-ketoconazole interaction in rats. *Xenobiotica*.

[13] Kremers P (2002). Can drug-drug interactions be predicted from in vitro studies?. *The Scientific World Journal*.

[14] Na DH, Ji HY, Park EJ, Kim MS, Liu KH, Lee HS (2011). Evaluation of metabolism-mediated herb-drug interactions. *Archives of Pharmacal Research*.

[15] Pao LH, Hu OY, Fan HY, Lin CC, Liu LC, Huang PW (2012). Herb-drug interaction of 50 Chinese herbal medicines on CYP3A4 activity in vitro and in vivo. *The American Journal of Chinese Medicine*.

[16] Lai MY, Hsiu SL, Hou YC, Tsai SY, Chao PDL (2004). Significant decrease of cyclosporine bioavailability in rats caused by a decoction of the roots of *Scutellaria baicalensis*. *Planta Medica*.

[17] Ueng YF, Shyu CC, Lin YL, Park SS, Liao JF, Chen CF (2000). Effects of baicalein and wogonin on drug-metabolizing enzymes in C57BL/6J mice. *Life Sciences*.

[18] Cho YA, Choi JS, Burm JP (2011). Effects of the antioxidant baicalein on the pharmacokinetics of nimodipine in rats: a possible role of P-glycoprotein and CYP3A4 inhibition by baicalein. *Pharmacological Reports*.

[19] Kronbach T, Mathys D, Umeno M, Gonzalez FJ, Meyer UA (1989). Oxidation of midazolam and triazolam by human liver cytochrome P450IIIA4. *Molecular Pharmacology*.

[20] Thummel KE, Shen DD, Podoll TD (1994). Use of midazolam as a human cytochrome P450 3A probe: II. Characterization of inter- and intraindividual hepatic CYP3A variability after liver transplantation. *Journal of Pharmacology and Experimental Therapeutics*.

[21] Gorski JC, Hall SD, Jones DR, Van Den Branden M, Wrighton SA (1994). Regioselective biotransformation of midazolam by members of the human cytochrome P450 3A (CYP3A) subfamily. *Biochemical Pharmacology*.

[22] Kobayashi K, Urashima K, Shimada N, Chiba K (2002). Substrate specificity for rat cytochrome P450 (CYP) isoforms: screening with cDNA-expressed systems of the rat. *Biochemical Pharmacology*.

[23] Ghosal A, Satoh H, Thomas PE, Bush E, Moore D (1996). Inhibition and kinetics of cytochrome P4503A activity in microsomes from rat, human, and cDNA-expressed human cytochrome P450. *Drug Metabolism and Disposition*.

[24] Ghosal A, Sadrieh N, Reik L, Levin W, Thomas PE (1996). Induction of the male-specific cytochrome P450 3A2 in female rats by phenytoin. *Archives of Biochemistry and Biophysics*.

[25] Zhang X, Galinsky RE, Kimura RE, Quinney SK, Jones DR, Hall SD (2010). Inhibition of CYP3A by erythromycin: in vitro-in vivo correlation in rats. *Drug Metabolism and Disposition*.

[26] Franke RM, Baker SD, Mathijssen RH, Schuetz EG, Sparreboom A (2008). Influence of solute carriers on the pharmacokinetics of CYP3A4 probes. *Clinical Pharmacology and Therapeutics*.

[27] Volak LP, Hanley MJ, Masse G (2013). Effect of a herbal extract containing curcumin and piperine on midazolam, flurbiprofen and paracetamol (acetaminophen) pharmacokinetics in healthy volunteers. *British Journal of Clinical Pharmacology*.

[28] Hoch M, Hoever P, Alessi F, Theodor R, Dingemanse J (2013). Pharmacokinetic interactions of almorexant with midazolam and simvastatin, two CYP3A4 model substrates, in healthy male subjects. *European Journal of Clinical Pharmacology*.

[29] Litterst CL, Mimnaugh EG, Reagan RL, Gram TE (1975). Drug metabolism by microsomes from extrahepatic organs of rat and rabbit prepared by calcium aggregation. *Life Sciences*.

[30] Jurica J, Dostálek M, Konecný J, Glatz Z, Hadasová E, Tomandl J (2007). HPLC determination of midazolam and its three hydroxy metabolites in perfusion medium and plasma from rats. *Journal of Chromatography B*.

[31] Tang Y, Zhu H, Zhang Y, Huang C (2006). Determination of human plasma protein binding of baicalin by ultrafiltration and high-performance liquid chromatography. *Biomedical Chromatography*.

[32] Kennedy DA, Seely D (2010). Clinically based evidence of drugherb interactions: a systematic review. *Expert Opinion on Drug Safety*.

[33] Mandlekar SV, Rose AV, Cornelius G (2007). Development of an in vivo rat screen model to predict pharmacokinetic interactions of CYP3A4 substrates. *Xenobiotica*.

[34] Kenworthy KE, Bloomer JC, Clarke SE, Houston JB (1999). CYP3A4 drug interactions: correlation of 10 in vitro probe substrates. *British Journal of Clinical Pharmacology*.

[35] Xing J, Chen X, Zhong D (2005). Absorption and enterohepatic circulation of baicalin in rats. *Life Sciences*.

[36] Li Y, Wang Q, Yao X, Li Y (2010). Induction of CYP3A4 and MDR1 gene expression by baicalin, baicalein, chlorogenic acid, and ginsenoside Rf through constitutive androstane receptor- and pregnane X receptor-mediated pathways. *European Journal of Pharmacology*.

[37] Hou YN, Zhu XY, Cheng GF (2000). Effects of baicalin on liver microsomal cytochrome P450 system. *Yao Xue Xue Bao*.

[38] Akao T, Kawabata K, Yanagisawa E (2000). Baicalin, the predominant flavone glucuronide of *Scutellariae radix*, is absorbed from the rat gastrointestinal tract as the aglycone and restored to its original form. *Journal of Pharmacy and Pharmacology*.

[39] Honig PK, Wortham DC, Zamani K, Conner DP, Mullin JC, Cantilena LR (1993). Terfenadine-ketoconazole interaction: pharmacokinetic and electrocardiographic consequences. *Journal of the American Medical Association*.

[40] Kupferschmidt HH, Ha HR, Ziegler WH, Meier PJ, Krähenbühl S (1995). Interaction between grapefruit juice and midazolam in humans. *Clinical Pharmacology & Therapeutics*.

[41] Lin JH, Lu AYH (2001). Interindividual variability in inhibition and induction of cytochrome P450 enzymes. *Annual Review of Pharmacology and Toxicology*.

[42] Tang C, Lin JH, Lu AYH (2005). Metabolism-based drug-drug interactions: what determines individual variability in cytochrome P450 induction?. *Drug Metabolism and Disposition*.

